# Determinants of contractual restraints in franchise contracting

**DOI:** 10.1002/mde.2961

**Published:** 2018-08-23

**Authors:** Ilir Hajdini, Aveed Raha

**Affiliations:** ^1^ Department of Business Administration, Faculty of Business, Economics, and Statistics University of Vienna Vienna Austria

## Abstract

Although an efficient design of franchise contracts requires from the franchisor to choose a bundle of contractual restraints as safeguarding and control mechanism, previous research has not explored the antecedents of contractual restraints as a bundle of contractual clauses. To address this gap, the aim of this study is to explain the determinants of the most important contractual restraints (i.e., exclusive dealing, exclusive territory, tying, resale price maintenance, call option, leasing, alienation, and noncompetition clauses), using transaction cost and relational governance reasoning. The regression results based on primary data from German and Swiss franchise systems provide support of hypotheses.

## 1 INTRODUCTION

Advantages of hierarchical control do not only result from integration or ownership but also from firms' ability to exercise decision control in exchange relationships (Heide, 1994; Stump & Heide, 1996; Weitz & Jap, 1995). According to Stinchcombe (1985), decision control as formal authority can be exercised between firms through contractual clauses. In franchising, formal authority in contracts refers to the clauses that restrain franchisees' activities in some desired manner (Lafontaine & Slade, 2014). For instance, by specifying geographical territories which define franchisees' local market boundaries, the franchisor can prevent horizontal externalities (Lafontaine & Slade, 2014); or by authorizing franchisees to acquire raw materials from the appointed suppliers and to sell only brand‐related products, the franchisor can influence the set and quality of products available to consumers (Marvel, 1982). Therefore, the efficient allocation of such restraints will influence the performance of franchise systems (Dutta, Heide, & Bergen, 1999).

With few exceptions, previous studies have not analyzed contractual restraints in networks (Dutta et al., 1999; Heide, 1994; Michael, 2000b). In franchising, scholars treat franchise contractual clauses either dichotomously (e.g., Dutta et al., 1999; Michael, 2000b; Schmidt, 1994) or holistically (Hendrikse, Hippmann, & Windsperger, 2015; Mooi & Ghosh, 2010; Solis‐Rodriguez & Gonzalez‐Diaz, 2012). The first approach fails to consider the possibility of association effects between contractual restraints. Although, the second approach that focuses on all possible contractual contingencies, for example, outlet hygiene, labor regulations, and business confidentiality, (Solis‐Rodríguez & González‐Díaz, 2017), hides the influence of the chosen antecedents on the contractual restraints (Poppo & Zenger, 2002; Reuer & Arino, 2007).

In this paper, we aim to empirically examine the antecedents of using contractual restraints from a bundle of the most important clauses in franchise contracts. More specifically, the study investigates the determinants of the franchisor's choice of contractual restraints of exclusive territory, exclusive dealing, tying and noncompetition arrangements, resale price maintenance, lease control, real option, and alienation rights (Dnes, 1993; Lafontaine & Slade, 2014; Schmidt, 1994; Windsperger, 2002). We argue that the franchisor's use of contractual restraints depends on transaction costs and relational variables, such as environmental uncertainty and trust. Transaction cost scholars have dominantly focused on explaining how business uncertainties could lead to more decision control (Williamson, 1975; Williamson, 1985; Williamson, 1991; Zhao, Luo, & Suh, 2004). However, they have not considered the role of relational aspects in interorganizational partnerships (Dyer & Singh, 1998; Poppo & Zenger, 2002; Zhang & Zhou, 2013). Less decision control can incentivize franchisees to acquire and transmit local market knowledge and improve partners' relations (Colombo & Delmastro, 2004; Gibbons, 2005; Gulati, 1995). Hence, the relationship between decision control and business uncertainties may be more complex than previously assumed.

The primary contribution of the study is to show that environmental uncertainty has a U‐shaped relationship with the number of contractual restraints included in franchise contracts. In particular, we contribute to transaction cost theory (TCT) by combining adaptation and control views of governance to argue that the franchisor incentivizes its franchisees to acquire and share local market information by using lower contractual control (i.e., fewer contractual restraints) under low to moderate levels of environmental uncertainty and realize transaction cost savings by using higher contractual control under high levels of uncertainty. In other words, we show that there is a trade‐off between the negative impact of contractual restraints on franchisees' local responsiveness under low to moderate uncertainty and the positive impact of contractual restraints on the franchisor control under high uncertainty. In addition, the franchisor's transaction‐specific investments and contract duration, which serve as safeguarding mechanisms, positively influence the use of contractual restraints in franchising. In contrary, franchisees' transaction‐specific investments, which serve as a bonding mechanism, negatively influence the use of contractual restraints in franchising. Furthermore, by applying relational governance reasoning, we show that the franchisor's trust as an informal governance mechanism negatively influences the degree of contractual restraints in franchising. Hence, this study also contributes to the literature of networks by showing that the relational view complements TCT to explain the franchisor's use of contractual restraints, which consequently may minimize the goal conflict among franchise partners (Nevin, 1995) and hence alleviate transaction costs and increase added value potential for end consumers. Thus, our findings are important for the management of franchise networks, because contract design is one of the main drivers of competitive advantage and franchise system performance (Azoulay & Shane, 2001; Shane, 1998).

The remaining structure of this study is organized as follows: In Section 2, the relevant contractual restraints in franchising are discussed. In Section 3, the hypotheses are developed. Subsequently, the data collection, methods, and results are presented. Discussion and conclusions follow.

## 2 OVERVIEW OF CONTRACTUAL RESTRAINTS IN FRANCHISE CONTRACTING

The most common form of restraints in retail contracts is the exclusive dealing clause (Lafontaine & Slade, 2014). It refers to a situation when franchisees agree not to offer services or products of competitors, thus dealing with the franchisor's single brand (Marvel, 1982). According to Scherer and Ross (1990), exclusive dealing assures a franchisor that its products or services will be sold with maximum effort. Further, Sass (2005) demonstrates that exclusive dealing impacts manufacturer–dealer conflict mitigation and social welfare enhancement. However, Heide, Dutta, and Bergen (1998) argue that, as exclusive dealing imposes costs on final customers, this may discourage firms from specifying such arrangements contractually.

The use of tying arrangements in franchise contracts implies a situation where the franchisor obliges franchisees to buy all or at least a proportion of their supplies from the franchisor or from its appointed suppliers. This contractual clause is mainly used to lower franchisors' monitoring costs, improve quality control, or protect goodwill (Michael, 2000b). For instance, Michael (2000b) show that although tying is not affected by market or outlet share, it is positively influenced by the equipment required and negatively affected by local advertising.

Exclusive territory assures franchisees that the franchisor will not establish new franchised or company‐owned outlets nearby the specified territory (Lafontaine & Slade, 2014). For instance, Ingram and Baum (1997) report that chain hotels in Manhattan are less successful in the presence of other operating units. Dutta et al. (1999) find that territorial restrictions reduce a manufacturer transaction costs from its distributor's free‐riding and information asymmetry when transaction‐specific investments are high. Kalnins (2004) finds that adding new units to franchised areas significantly reduces the revenues of other incumbent units, whereas this is not the case for company‐owned outlets. To determine whether the exclusivity clause mitigates encroachment conflicts for both franchise partners, Nair, Tikoo, and Liu (2009) formulate an analytical model that supports this view.

Price restrictions in franchise chains occur when the franchisor gains control over the chain's pricing strategy, recommending either minimum floors or maximum ceilings (Perry & Besanko, 1991). Such a contractual arrangement prevents intrabrand price competition, thus encouraging the franchisor to undertake intangible advertising and marketing investments (Rey & Verge, 2008; Schmidt, 1994; Windsperger, 2002). Blair and Lafontaine (1999) examine whether restricting prices violate antitrust regulations. They report that limiting the maximum prices would incentivize franchisees to compete through other means and thereby enhance consumer welfare.

To encourage a more entrepreneurial relationship, the franchisor offers the lease option to franchisees instead of a tenancy. The lease right serves as a hostage, that is, a means by which the franchisor can control franchisees and reduce monitoring costs, as franchisees can be asked to move out and return their valuable context‐specific investments to the leaseholder, that is, the franchisor (Dnes, 1992, 1993).

With the call option clause, the franchisor obtains the legal right to acquire franchisee‐owned outlets at the termination of the contract. In addition to the strategic value creation function, an explicit call option specified in franchise contracts has a safeguarding/control function (Trigeorgis & Reuer, 2017). Under the call option right, the franchisor can better protect its transaction‐specific investments from potential franchisee opportunistic behavior (Reuer & Tong, 2005).

The noncompetition covenant obliges franchisees not to undertake the same business activities within their former franchise territories for a certain period (Dnes, 1993). Restrictions on post‐contract competition can deter franchisees from free‐riding behavior (Dnes, 2008).

In sum, we can conclude that the contractual restraints are efficiency‐enhancing mechanisms in franchising networks. In the following, we examine the antecedents of using contractual restraints as a bundle of contractual clauses.

## 3 DETERMINANTS OF CONTRACTUAL RESTRAINTS

### 3.1 Transaction cost hypotheses

TCT is an important theoretical perspective for the empirical analysis of contracts (Hendrikse et al., 2015; Lafontaine & Slade, 2014) by highlighting that certain types of transaction attributes (e.g., transaction‐specific investments and uncertainties) are predictors of the choice of contract terms and governance structures (Klein, Crawford, & Alchian, 1978; Williamson, 1975; Williamson, 1985).

#### 3.1.1 Environmental uncertainty

Uncertainties result from the inability of partners to specify all exchange contingencies in contracts ex ante or to evaluate performance ex post. This view assumes that economic actors are bounded rational (Williamson, 1985). The majority of scholars agree that environmental uncertainty including institutional, cultural, or economic factors aggravate the problem of bounded rationality in designing more complete contracts, which, in turn, influences transaction costs and the design of firms' governance structures (Gulati, Lawrence, & Puranam, 2005; Rindfleisch & Heide, 1997; Williamson, 1975; Williamson, 1991). Specifically, in response to increased transaction costs due to high environmental uncertainty, TCT predicts that firms increase control by vertical integration. However, not all transaction cost proponents agree on the direction of influence that environmental uncertainty has on firms' governance structures (Geyskens, Steenkamp, & Kumar, 2006; Klein, 1989; Walker & Weber, 1984). As Klein (1989: 256) states regarding uncertainty, “… different facets of it lead to both a desire for flexibility and a motivation to reduce transaction costs.” As a result, we argue that two views of governances should be considered simultaneousely: the adaptation view and the control view of governance.

The adaptation view argues that the franchisor needs to adapt standardized business formats to the specifics of the local market environment (Gulati et al., 2005). This view encompasses the need to adapt contract design to the needs of franchise partners serving as an adjustment mechanism to increase franchisees incentives for information acquisition and sharing (Gibbons, 2005). Given that the level of dependencies between the network partners is relatively high, more information sharing and communication are required to protect such relationships against exchange hazards (Williamson, 1991). Accordingly, this view ascertains that more autonomy under environmental uncertainty creates information‐processing benefits and reduces leaks and delays in transmitting the flow of information between the network partners (Colombo & Delmastro, 2004; Zabojnik, 2002). Thus, in the presence of environmental unpredictability, partners are willing to remain more flexible (Balakrishnan & Wernerfelt, 1986; Klein, 1989; Teece, 1992). Flexibility can be obtained if less formal authority is exercised through fewer contractual restraints.

Contrary to the above approach, the control view of governance argues that, under high environmental uncertainty, firms cope more effectively with uncertainty by increasing the level of control (Williamson, 1975). The control rights are more specified in contracts which serve to regulate exchanges of supplies, monitor quality, propose maximum price levels, and set sales targets, among other factors. Stinchcombe (1990) states that firms specify more elements of hierarchical control in business relations under a higher level of environmental uncertainty. Similarly, Noordewier, John, and Nevin (1990) show that environmental uncertainty is positively related to the level of control in interfirm alliances. Thus, the extent of contingencies that could weaken the franchisor–franchisee relationship grows when the environment becomes more uncertain, which, in turn, increases the need for more contractual safeguards (Williamson, 1975, Williamson, 1985). Such safeguarding restraints, even if costly to enforce and rarely exercised under conditions of high environmental uncertainty, increase the positive impact of “unexercised threats” (Bradach, 1998) and thereby reduce transaction costs and preserve the franchisor's transaction‐specific investments.

Consequently, accounting for both views, we argue that there is a U‐shaped relationship between environmental uncertainty and the degree of contractual restraints used in franchise contracts. In compliance with the adaptation view, a lower degree of contractual restraints will increase franchisees' incentives for information acquisition and knowledge sharing. However, as the level of uncertainty increases, the benefits of specifying a higher number of restraints in contracts exceed the benefits from franchisees' incentives for local information acquisition and sharing. Hence, in the presence of high environmental uncertainty, transaction costs are exacerbated due to the difficulty of controlling, verifying, or monitoring potential franchisees opportunism, for example, free riding on the quality of products or services (El Akremi, Mignonac, & Perrigot, 2011). Consequently, under increasing environmental uncertainty, we argue that there is a *trade‐off* between the local responsiveness effect of contracts with fewer restraints and the transaction cost savings effect of contracts with more restraints. Under low to moderate levels of environmental uncertainty, the franchisor will less likely include contractual restraints to incentivize franchisees' entrepreneurial responsiveness in the local market. However, under high environmental uncertainty, the value‐increasing effect of more contractual restraints due to transaction cost savings under higher contractual control exceeds the residual income benefits of local responsiveness under fewer contractual restraints. Hence, an alternative hypothesis can be formulated:

Hypothesis 1 There is a U‐shaped relationship between environmental uncertainty and the degree of contractual restraints in franchise contracts.

#### 3.1.2 Transaction‐specific investments

The second transaction cost dimension is transaction‐specific investments. Assets which are tailored to a specific partnership include tangible assets, for example, business premises, facilities, and equipment, along with intangible assets, such as training and human capital (Anderson & Weitz, 1986; Watson, Stanworth, Healeas, Purdy, & Stanworth, 2005). When a franchise network starts expanding, the franchisor needs to undertake important specific investments to reap the rewards of relationship‐specific quasi‐rents (Katz, 2008). Such investments might be threatened by potential opportunistic franchisees, as specific assets cannot be easily redeployed beyond the relationship (Hendrikse et al., 2015; Williamson, 1975). Thus, asset specificity encourages the franchisor to assign more contractual restraints that mitigate opportunism.

However, in situations where the quasi‐rent stream due to franchisees' transaction‐specific investments exceeds the gains from opportunism, the franchisor might design contracts by specifying fewer contractual restraints in response to joint relationship‐specific investments (Hendrikse et al., 2015). This occurs when franchisees' transaction‐specific investments signal that opportunistic behavior is unlikely to occur (Michael, 2000a). In other words,

Hypothesis 2a Franchisor's transaction‐specific investments will positively influence the degree of contractual restraints in franchise contracts.

Hypothesis 2b Franchisee's transaction‐specific investments will negatively influence the degree of contractual restraints in franchise contracts.

#### 3.1.3 Contract duration

TCT predicts that in a long‐term exchange relationship, the franchisor attempts to identify future contingencies and to prevent contractual hazards by incorporating more contractual safeguards (Klein et al., 1978; Williamson, 1975, 1985). Hence, longer contracts require more contractual restraints that are used to attenuate future conflicts and enhance partners' commitment (Joskow, 1985; Williamson, 1985). According to Joskow (1987), p. 169) “A long‐term contract that specifies the terms and conditions for some set of future transactions ex ante, provides a vehicle for guarding against ex post performance problems.” Hence, this can lead to reduced ambiguity about franchise partners' contractual obligations and enhances their incentives for specific investments and thereby lowers the likelihood for opportunism. Therefore,

Hypothesis 3 Contract duration will positively influence the degree of contractual restraints in franchise contracts.

### 3.2 Relational governance hypotheses

TCT perspective is limited in explaining franchise relationships as it neglects the dimension of relational complexity within franchise systems (Pizanti & Lerner, 2003). The relational governance approach argues that trust, norms, and solidarity are particularly important for sustaining exchange relationships (Dyer & Singh, 1998; Heide & John, 1992; Poppo & Zenger, 2002; Zhang & Zhou, 2013). Specifically, trust increases cooperation, reduces the negative effects of conflicts (Anderson & Narus, 1990), and dilutes agency problems in franchise networks (Cochet & Garg, 2008). Further, trust facilitates strategic changes carried out by the franchisor (Croonen, 2010), increases franchisees' satisfaction (Dickey, Harrison McKnight, & George, 2008), and improves the level of franchisee‐compliant behavior (Davies, Lassar, Manolis, Prince, & Winsor, 2011; Dickey et al., 2008). Hence, as contracts cannot specify all contingencies, governance based on trust can reduce opportunism, mitigate transaction costs, and increase satisfaction.

Previous research has used different concepts and measures to capture the construct of trust (Das & Teng, 2004; Ganesan, 1994; Malhotra & Murnighan, 2002; McAllister, 1995). In organization theory, there is a differentiation between general trust and experience‐based trust (Hendrikse et al., 2015). General trust refers to a situation in which an individual has a positive trustworthy attitude toward others by default, even with no previous interactions (Yamagishi & Yamagishi, 1994). Prior to partnership initiation, if franchise partners hold a high level of trust, they also tend to expect lower relational risks (Das & Teng, 2004; Griessmair, Hussain, & Windsperger, 2014). From the franchisor perspective, this implies that high levels of general trust lead to the perception of lower relational risks and results in choosing fewer contractual restraints in franchise contracts. Formally,

Hypothesis 4a General trust will negatively influence the degree of contractual restraints in franchise contracts.

As supported by the interorganizational network literature, successive collaborations between network partners can lead to trust and interorganizational routines (Dyer, 1997; Gulati, 1995; Uzzi, 1997; Zollo, Reuer, & Singh, 2002). Franchise partners' prior positive experience signals credibility and trust (Anderson & Weitz, 1989). Further, repeated interactions facilitate and improve the mutual understanding of partners' managerial expectations, organizational culture, and operations (Gulati, 1995). Therefore, trust developed through previous interactions lessens the potential for post‐contract transaction costs, such as monitoring and dispute resolution. Consequently, the franchisor's trust based on prior experience regarding its franchisees reduces the need for formal restraints in franchise contracts (Reuer & Arino, 2007). Correspondingly,

Hypothesis 4b Experience‐based trust will negatively influence the degree of contractual restraints in franchise contracts.

## 4 EMPIRICAL ANALYSIS

### 4.1 Data collection

To test these hypotheses, data were collected from the franchise systems in Germany and Switzerland. To refine and improve the questionnaire, several interviews were conducted with franchise professionals from the respective franchise associations. Twenty franchisors in Austria participated in the final modification process. They suggest that to ensure a more confident level of knowledge about the franchise partners, questionnaires should aim at respondents with a minimum number of years of interorganizational experience and a minimum network size (e.g., number of outlets). Thus, it was decided that any system selected should have started franchising at least 2 years prior to the study and should have at least five operating outlets to be considered a useful observation. As a result, out of 1,013 systems listed by the German Franchise Federation and the Swiss Franchise Association, the questionnaire was mailed to 667 German and Swiss franchise systems.

The questionnaires were sent to senior managers who were considered responsible for franchise expansion, based on their expertise and relevance to the subject under investigation (McKendall & Wagner III, 1997). The number of questionnaires returned was 166, representing a response rate of approximately 28% from Germany and 17% from Switzerland. However, due to missing values, 116 responses are used for the analysis. To trace the likelihood of nonresponse bias, the results obtained from both late and early respondents were examined to see if they were significantly different (Armstrong & Overton, 1977). The late respondents, which include those who completed the questionnaire 4 weeks after the first group of respondents, served as proxies for nonrespondents. The analysis of variance test revealed no significant difference between the two respondent groups.

Further, the common method variance was tested by conducting a single factor analysis of the items of all subjective measures, which revealed three distinct factors with an eigenvalue greater than 1.0. The first component explains 23.11% of the variance, whereas all of them taken together explain 64.61% (Anderson & Gerbing, 1988). Thus, Harman's single‐factor test negates the possibility of confounded interpretations since no factor accounts for most of the variance.

### 4.2 Measures

#### 4.2.1 Dependent variable

Contractual restraints represent the variable of interest. The most important contractual restraints used in franchise contracting were utilized in this study, such as exclusive territory, exclusive dealing, tying and noncompetition arrangements, resale price maintenance, lease control, real option and alienation rights. Franchisors were asked to indicate whether they use restraint clauses in their contracts (1 if yes, 0 otherwise). Table 1 contains the relative frequency of contractual clauses that restrain the cooperation between the franchise partners. The exclusive territory clause is most frequently used, followed by the exclusive dealing clause, whereas lease control is the least frequently applied contractual restraint.

**Table 1:** The allocation of contractual restraints in franchise contracts

No.	Restraint clauses	Presence	Frequency	Percent
1	Dealing	no	47	30.72
		yes	106	69.28
2	Tying	no	71	46.41
		yes	82	53.59
3	Resale price	no	63	41.72
	maintenance (nonbinding)	yes	88	58.28
4	Territory	no	39	25.66
		yes	113	74.34
5	Leasing	no	81	52.94
		yes	72	47.06
6	Call option	no	56	37.09
		yes	95	62.91
7	Alienation	no	59	38.82
		yes	92	60.53
8	Noncompetition	no	57	37.50
		yes	95	62.50

*Note*. “Yes” means that the contractual restraint was included in the contract.

The operationalization of this measure follows that used by Parkhe (1993) and Reuer and Arino (2007). Hence, the different types of franchise restraints are arrayed in increasing order of strength or severity to arrive at a global measure that represents the degree of franchise contractual restraints that are in use. Contractual restraint stringency is derived using the following weighting scheme:

(1) $$\text{DCRF}\;\left(\text{weighted}\right)=\frac{1}{36}\sum_{i=0}^{8}A_{i},$$

where DCRF represents the degree of used contractual restraints in franchising, *A*_*i*_ equals *i* if the *i*th restraint is employed, and zero if not (Parkhe, 1993). That is, *A*_*i*_ equals one if the first restraint is used (two if the second is used) and zero otherwise. The sum ranges between 0 and 36, which when divided by 36, yields values between 0 and 1. In other words, all eight restraints are used if DCRF equals 1.

The alternative operationalization is to use an unweighted measure of the DCRF as the dependent variable. Although assuming equivalent stringency of individual restraints, this approach tests whether the weighting scheme plays a role in estimating the importance of individual covariates on the dependent variable. The unweighted measure takes the following form:

(2) $$\text{DCRF}\;\left(\text{unweighted}\right)=\sum_{i=0}^{8}B_{i},$$

where Bi equals 1 if the ith restraint was employed and zero otherwise. The unweighted dependent variable is estimated using ordered probit models ranging from 0 to 8.

Finally, we additionally applied a dichotomous categorization on the dependent variable, where the variable is coded 1 if more than half of the restraints are assigned and 0 otherwise. The dichotomous dependent variable is estimated using probit models ranging from 0 to 1. Assuming equivalent stringency of individual restraints, this approach should increase the explanatory power of individual covariates on the dependent variable given its dummy categorization and thus more clearly reveal the influence of the chosen antecedents. Thus,

(3) PrDCRF=1∣X=ΦX′β;DCRF=1,136∑i=08Ai>τ0,136∑i=08Ai≤τ,

where τ equals the threshold of four restraints.

#### 4.2.2 Independent variables

Except for contract duration, prior experience, and franchisees' transaction‐specific investments, all other explanatory constructs were assessed via multi‐item measures. Franchisors were asked to rate all items on a 7‐point Likert scale (see Appendix A).

*Environmental uncertainty* was adopted from Celly and Frazier (1996) and John and Weitz (1988). We asked franchisors to assess three items based on 7‐point Likert scale regarding their possibility to forecast development and fluctuations of outlet sales, the unpredictability of the local market, and volatility of the local economic situation. The reliability of this measure was assessed using Cronbach's α (α = 0.748) and, following confirmatory factor analysis, the average variance extracted (AVE = 0.65).

*Franchisors' transaction‐specific investments* are measured as follows: Franchisors were asked to rate their specific investments based on 7‐point Likert scale about their expenses for the franchisees' training, the setup of the local outlet, and the technical support to the franchisees at the beginning of the relationship (α = 0.64, AVE = 0.58). *Franchisees' transaction‐specific investments* are measured by the average investments required to open a franchised outlet (Dnes, 1993). *Contract duration* refers to the number of contract years; on average franchisees are initially granted close to 7‐year contracts.

The measures used to test the relational governance hypotheses (H4a/H4b) are general trust and prior experience as a proxy for experience‐based trust. Similar to previous studies on general trust (e.g., Lazzarini, Miller, & Zenger, 2008; Yamagishi & Yamagishi, 1994), franchisors were asked to rate the following items based on 7‐point Likert scale: Most people trust others, most people are trustworthy, and most people behave cooperatively if they are trusted (α = 0.80, AVE = 0.54). Following Gulati (1995) and Reuer and Arino (2007), *prior experience* was measured with the number of years because the franchisor first started to cooperate with their franchise partners.

#### 4.2.3 Control variables

To account for possible confounders, we initially control for size, which represents the total number of franchised and company‐owned outlets. Smaller firms might lack the necessary resources and capabilities to craft sophisticated contractual agreements (Reuer & Arino, 2007). Further, larger firms may have a higher control capacity (Erramilli & Rao, 1993). We also incorporated a dummy variable to control for sectoral effects (0 for service firms, 1 for product franchising firms). Intangible assets (e.g., local market know‐how, knowledge transfer, monitoring capabilities) vary between different sectors. Service franchising firms might need more intangible assets compared with product franchising firms (Zeithaml, Parasuraman, & Berry, 1985). Finally, multiunit franchising (MUF) allows a franchisee to open two or more outlets at specified geographical territories under the same franchise brand. Hence, the franchisor may need to make more context‐specific investments tailored to the broader geographical area of its multiunit franchisees. In order to safeguard such additional specific investments against potential opportunism, the franchisor might specify more contract terms. To control for the effect of MUF, we derived this variable as a ratio of franchised outlet to the number of franchisees (Gomez, Gonzalez, & Vazquez, 2010; Hussain & Windsperger, 2013).

## 5 RESULTS

Descriptive statistics and Pearson correlation coefficients are reported in Table 2. Because few variables (e.g., size, contract duration, or prior experience) show high standard deviations or nonnormal distributions, natural logarithms were employed in OLS estimations. In addition, variance inflation factors (ranges from 1.13 to 8.36) indicate that multicollinearity does not affect the results of our analysis.

**Table 2:** Descriptive statistics and correlations

	Variables	Mean	*SD*	1	2	3	4	5	6	7	8	9	10
1.	Contractual restraints(unweighted)	4.70	1.47										
2.	Contractual restraints (weighted)	.50	.18	.84***									
3.	Sector	.59	.49	−.06	.13								
4.	Size	137	303	−.10	−.17**	−.17**							
5.	Multi‐unit franchising	19.6	24.8	.28**	.29***	−.02	.25**						
6.	Environmental uncertainty	3.66	1.38	−.10	−.14*	.01	−.04***	−0.27***					
7.	Franchisor specific investments	13.0	3.88	.27***	.31***	−.10	−.05*	.15*	.03				
8.	Franchisee specific investments	10.5	1.87	.06	.03	−.06	.13***	.45***	−.21***	.31			
9.	Contract duration	6.69	3.24	.21**	.19**	.07	.03***	.38***	−.25**	.18***	.50		
10.	General trust	4.44	1.31	−.19**	−.13	.11	.06**	−.19**	.23	.06	−.08	.00	
11.	Experience‐based trust	11.22	8.49	−.02	−.15*	−.08	.53***	.28***	.04	−.08*	.15**	.17	−.07

*** *p* < 0.01;

** *p* < 0.05;

* *p* < 0.1.

The multivariate analysis is presented in Table 3. The discrete distribution of all contractual restraints captured by the unweighted dependent variable (i.e., Equation (2)) is estimated applying ordered probit estimation presented in models 1 and 2. The subsequent models 3 and 4 are estimated applying OLS estimation method as the dependent variable of contractual restraints is weighted (i.e., Equation (1)). Models 5 and 6 are estimated applying probit regressions for the underlying dependent variable. The objective is to test whether dichotomous categorizations and whether stringency weights applied to the dependent variable reveal additional information on the influence of selected antecedents. The likelihood ratios in ordered probit and probit models and the F statistic in OLS estimations indicate the joint significance of the included theoretical antecedents when compared with the baseline models.

**Table 3:** Regression results from multivariate analysis^a^

Determinants of the degree of contractual restraints in franchising contracts							
	Ordered probit, DCRF (unweighted)		OLS, DCRF (weighted)		Probit, DCRF (weighted)	
Variable	M 1	M 2	M 3	M 4	M5	M6
Intercept(s)	‐‐	‐‐	6.94 (1.35)	6.92 (1.33)	1.75 (1.06)	2.56 (1.66)
Sect	−.12 (.26)	−.07 (.20)	−.22 (.24)	−.22 (.24)	.43 (.25)	.46 (.29)
Size	−.00 (.00)	.00 (.00)	.01 (.09)	.01 (.09)	.00 (.00)	.00 (.00)
Multiunit franchising	.01^**^ (.00)	.01** (.00)	.01** (.00)	.01** (.01)	.01* (.00)	.01* (.00)
Fixed effects	−.00 (.00)	.00 (.00)	−.01 (.00)	.00 (.01)	.00 (.00)	.00 (.00)
H1: Environmental uncertainty	−1.7*** (.42)	−1.6** (.41)	−1.7*** (.01)	−1.3** (.58)	−2.2*** (.76)	−2.6*** (.83)
H1: (Environmental uncertainty)^2^	.21*** (.05)	.20*** (.05)	.20*** (.06)	.16*** (.07)	.26*** (.09)	.31*** (.10)
H2: Franchisor's investments	.05** (.02)	.05*** (.02)	.08*** (.03)	.08*** (.03)	.12*** (.03)	.12*** (.03)
H2: Franchisees' investments	−.00* (.00)	−.00 (.00)	−.20* (.10)	−.21** (.10)	−.00*** (.00)	−.00*** (.00)
H4: Contract duration	.06* (.04)	.08** (.04)	.51* (.29)	.71*** (.30)	.09* (.05)	.12** (.05)
H6: General trust		−.14** (.07)		−.27** (.13)		−.04 (.11)
H5: Experience‐based trust		−.01 (.01)		−.22* (.12)		−.04** (.01)
χ2 (*df*)/F	69 (9)	74 (12)	6.7***	7.0***	39 (9)	43 (11)
Likelihood ratio (B)	−183.1	−178.6			−62.89	−58.59
Correct classification					76%	77%
Pseudo *R* ^2^/Adj *R* ^2^	.133	.142	.289	.330	.258	.297

*Note*. *N* = 116. Standard errors are shown in parentheses.

*** *p* < 0.01;

** *p* < 0.05;

* *p* < 0.1.

**Figure 1:**
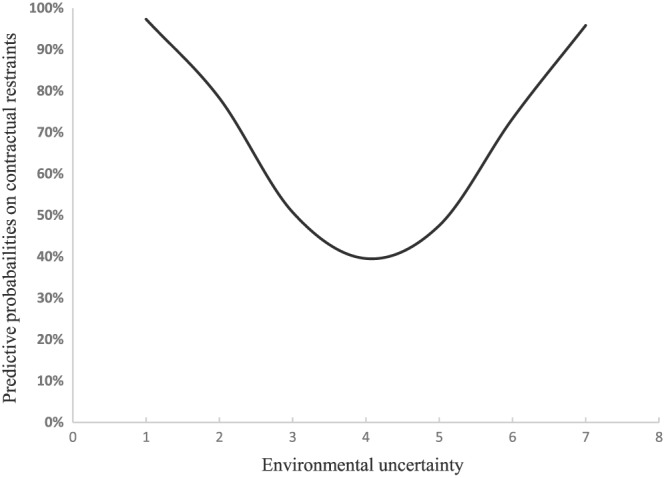
U‐shaped relationship between the degree of contractual restraints and environmental uncertainty

The first hypothesis envisages that there is a U‐shaped relationship between the degree of specified restraints in franchising contracts and environmental uncertainty. The empirical results on the aggregated contractual restraints support this prediction. This relationship implies that the likelihood of specifying more contractual restraints is first decreasing and then increasing with rising environmental uncertainty. The evidence supports H1 that up to a certain level of uncertainty, the franchisor strengthens the franchisees' motivation to acquire and share information about the local market and increase their responsiveness to promptly react to customers' claims at the local market. However, with rising environmental uncertainty, the franchisor's residual income increasing effect of local responsiveness is more than compensated by the transaction cost savings of higher control based on more contractual restraints. Figure 1 depicts marginal effects that an increase in the level of environmental uncertainty has on the probability of using more contractual restraints (while accounting for the effect of all other variables).

The second hypothesis (2a) that predicts a positive influence of the franchisor's transaction‐specific investments on the degree of specified contractual restraints is supported by all models. To safeguard the franchisor's specific investments from potential opportunism, franchise contracts contain more contractual restraints (Hendrikse et al., 2015). However, the prediction that franchisees' specific investments (2b) will negatively impact the degree of specified restraints in franchise contracts is weakly supported in the models M4, M5, and M6. This might be explained by the fact that the franchisor with strong brand reputation may exercise more bargaining power to standardize and formalize contracts (Kidwell, Nygaard, & Silkoset, 2007).

The hypothesis that the contract duration (3) positively influences the number of contractual restraints is also empirically supported. As long‐term cooperation increases the unpredictabilities about future environmental or behavioral contingencies, the franchisor will specify more contractual restraints. Finally, consistent with the predictions of the relational governance view (4a and 4b), our data provide support for the hypotheses that trust, and prior experience negatively influence the specification of explicit contractual restraints in franchise networks. Negotiation, monitoring, and enforcement costs are lower in relationships between network partners with higher trust (Das & Teng, 2004).

Moreover, the results show that when the franchisor employs MUF, it specifies more restraints to safeguard its greater specific investments. The investigation of clauses applying alternative weighting schemes for the stringency of restraints and the dichotomous categorization of the dependent variable reveals the robustness of the results (see Table 3).

## 6 DISCUSSION AND IMPLICATIONS

Despite the growing evidence that decision control and contractual restrictions influence the performance and survival of franchise systems (Azoulay & Shane, 2001; Hajdini, Klapper, Rommer, & Windsperger, 2017; Shane, 1998), previous research has not explored the antecedents of the most important contractual restraints as a bundle of contractual clauses in franchising (Cochet & Garg, 2008; Dutta et al., 1999). To address this gap, the aim of this study was to explain the determinants of contractual restraints in franchise contracting by using transaction cost and relational governance reasoning.

The study provides evidence for a U‐shaped relationship between environmental uncertainty and the degree of specified contractual restraints, thereby contributing to TCT literature. More precisely, combing adaptation and control views of governance, we showed that the franchisor is more likely to reduce the number of restraints to increase franchisees' motivation for information acquisition and sharing when there is a low to moderate environmental uncertainty. However, when the level of uncertainty increases, the economic value‐increasing effect of specifying a higher number of restraints exceeds the incentive‐related benefits due to franchisees' higher motivation for information acquisition under fewer contractual restraints. Further, under conditions of high environmental uncertainty, the inclusion of additional contractual restraints can increase the effect of contractual threats and reduce transaction costs (Bradach, 1998; El Akremi et al., 2011).

Consistent with the TCT (Williamson, 1985), this study shows how franchise partners' transaction‐specific investments and the length of contract influence the degree of specified restraints in franchise contracting. More specifically, we complement prior studies on individual restraints by demonstrating that from the bundle of eight contractual clauses the franchisor uses more restraints for safeguarding purposes. In addition, the empirical results indicate that in response to high transactions costs associated with long‐term contracts, the franchisor uses more contractual restraints.

Although there is evidence in the relational governance literature that trust is an important informal governance mechanism in various interorganizational configurations (Dyer & Singh, 1998; Gulati, 1995; Heide & John, 1992), this study demonstrates that the relational view complements the TCT perspective to explain the franchisor's choice of contractual restraints. The results indicate that trust as a relational norm can serve as an informal control mechanism that reduces the requirement to use more formal restraints in franchise contracts. This can be explained by the fact that when the franchisor has a high level of general and experience‐based trust, it expects lower relational risks (Das & Teng, 2004; Griessmair et al., 2014).

The results of the study have important implications for the design of franchise contracts. First, if uncertainties are perceived as high and the franchisor's transaction‐specific investments are high relative to the franchisees', the franchisor uses more contractual restraints to utilize their efficiency‐enhancing properties and safeguard its transaction‐specific investments. However, the franchisor has to consider that franchisees are incentivized under a lower level of contractual control to acquire local know‐how and react more flexibly to local customer needs. Second, this study shows that general and experience‐based trust reduce the requirement of formal contractual restraints. Hence, the franchisor may provide franchisees with more autonomy (i.e., less contractual restraints) when general and experience‐based trust are high. This enables the franchisor to realize transaction cost savings in contract design, direct control, and monitoring mechanisms.

The study has two important limitations. Future research may investigate which contractual restraints are in favor of the franchisor and which are in favor of franchisees to explain the efficient bundling of contractual restraints. In addition, this study has not examined the performance outcome of contractual restraints; hence, future research may focus on performance consequences of different bundles of contractual restraints in franchising networks.

Overall, this is the first empirical study that examines the determinants of contractual restraints from a bundle of clauses in franchise contracts. Based on TCT and relational governance views, we develop a new model that explains the conditions under which efficiency‐enhancing contractual restraints are specified in franchise contracts. In addition, this study adds to the literature of relationships between formal and relational governance mechanisms by showing that trust is an important behavioral prerequisite to use fewer contractual restraints in franchise contracts (Poppo & Zenger, 2002; Ryu, Min, & Zushi, 2007).

## APPENDIX A

**Table A1:** Measures of the variables

Constructs	Measurement	Items	
Contractual restraints	Dichotomous; Reuer and Tong (2005)	Do you (the franchisor) include the following clauses in the franchise contract:	
		1. Tying arrangement: Franchisees must purchase more than 50% of input from you (franchisor) or your appointed supplier(s).	
		2. Exclusive dealing clause: Franchisees may sell products other than those of the franchisor (reversed).	
		3. Resale price maintenance: You set the nonbinding prices for franchisees' offered products/services.	
		4. Exclusive territory clause: The market area of franchisees is specified geographically.	
		5. Lease control: You are the owner or tenant of the location of the franchised outlet.	
		6. Call option clause: You have a contractual option to buy the franchise operation at contract termination or potentially sold by your franchisees.	
		7. Alienation right: The franchise operation is heritable (reversed).	
		8. Noncompetition clause: The franchise agreement contains a clause for the competitive activities after the expiry/termination of the contract.	
Franchisor's transaction‐specific investments α = 0.64 AVE = 0.58	7‐point items, anchored by “Strongly disagree” [1] to “Strongly agree” [7], adopted from Heide and John (1990) and Geyskens et al. (2006).	To what extent do you bear the expenses for the following:	
		1. Train franchisees at the beginning of the contractual relationship	
		2. Technical support provided to franchisees at the beginning of the contractual relationship	
		3. Setting‐up local outlets	
Franchisee's transaction‐specific investments	Adapted from Dnes (1993)	Specify the average investments required by a franchisees to build a new franchised outlet (in Euros)	
Environmental uncertainty α = 0.75 AVE = 0.65	7‐point items, anchored by “Strongly disagree” [1] to “Strongly agree” [7], adapted from Celly and Frazier (1996) and John and Weitz (1989).	To what extent do you agree with the following: 1. Sales at the local outlets are very fluctuating; 2. It is very difficult to forecast the market development in the local markets; 3. Economic environment is changing rapidly in the local markets.	
General trust α = 0.80 AVE = 0.54	7‐point items, anchored by “Strongly disagree” [1] to “Strongly agree” [7], adapted from Yamagishi and Yamagishi (1994) and Lazzarini et al. (2008).	To what extent do you agree with the following: 1. Most people trust others;	
		2. Most people are trustworthy;	
		3. Most people behave cooperatively if they are trusted.	
Experience‐based trust	Adapted from Reuer and Arino (2007)	Specify the number of years since you first started to cooperate with your franchise partners
Contract duration	Adapted from Vázquez (2007).	Specify the number of contract years you offer to your franchisees
Size		Specify the number of employees in your headquarters
Sector		Which industry does your business belong to?—Service franchising—Product franchising

*Note*. α = Cronbach's alpha; AVE = average variance extracted.
